# Deregulation of UBE2C-mediated autophagy repression aggravates NSCLC progression

**DOI:** 10.1038/s41389-018-0054-6

**Published:** 2018-06-13

**Authors:** Jiwei Guo, Yan Wu, Jing Du, Lijuan Yang, Weiwei Chen, Kaikai Gong, Juanjuan Dai, Shuang Miao, Dan Jin, Sichuan Xi

**Affiliations:** 1 Cancer Research Institute, Binzhou Medical University Hospital, 256603 Binzhou, P.R. China; 2 Department of Pain Ward, Binzhou Medical University Hospital, 256603 Binzhou, P.R. China

## Abstract

The roles of aberrantly regulated autophagy in human malignancy and the mechanisms that initiate and sustain the repression of autophagy in carcinogenesis are less well defined. Activation of the oncogene UBE2C and repression of autophagy are concurrently underlying the initiation, progression, and metastasis of lung cancer and exploration of essential association of UBE2C with autophagy will confer more options in searching novel molecular therapeutic targets in lung cancer. Here we report that aberrant activation of UBE2C in lung tumors from patients associates with adverse prognosis and enhances cell proliferation, clonogenicity, and invasive growth of NSCLC. UBE2C selectively represses autophagy in NSCLC and disruption of UBE2C-mediated autophagy repression attenuates cell proliferation, clonogenicity, and invasive growth of NSCLC. Autophagy repression is essentially involved in UBE2C-induced cell proliferation, clonogenicity, and invasive growth of NSCLC. Interference of UBE2C-autophagy repression axis by Norcantharidin arrests NSCLC progression. UBE2C is repressed post-transcriptionally via tumor suppressor miR-381 and epitranscriptionally stabilized with maintenance of lower m^6^A level within its mature RNAs due to the upregulation of m^6^A demethylase ALKBH5 in NSCLC. Collectively, our results indicated that deregulated UBE2C-autophagy repression axis drives NSCLC progression which renders varieties of potential molecular targets in cancer therapy of NSCLC.

## Introduction

Elusive carcinogenesis and ineffective therapeutic for non-small cell lung cancer (NSCLC) remain the major obstacle in reducing the lung cancer-related deaths globally so far^1–3^. Limited improvements in diagnosis and therapeutic options still left most lung cancer patients suffering recurrence within 5 years lacking more effective targeted therapy^2,3^. The precise molecular characterization of the key aberrantly deregulated signal cascades in initiating and maintaining the lung carcinogenesis and progression is fundamentally required in finding novel molecular targets of NSCLC. Ubiquitin-conjugating enzyme E2C (UBE2C), one of ubiquitination enzymes catalyzing degradation of proteins into smaller polypeptides, amino acids, and ubiquitins in 26S proteasome^4,5^, is involved in carcinogenesis via regulating cell cycle, apoptosis, and transcriptional process^6–8^, in which UBE2C was upregulated and correlated with poorer overall survival (OS) and progression-free survival of NSCLC patients^7^. UBE2C overexpression enhanced tumor cell growth and colony formation in malignant transformation in vitro and in vivo^9^.

Programmed cell death (PCD) involves the activation of catabolic enzymes in the formation of apoptotic bodies^10–12^ and begins with a catabolic process that assembles autophagosomes and merges them with lysosomes for recycling and degradation^13,14^. As the subtypes of PCD, both apoptosis and autophagy are UBE2C-targeted cellular phenotypes in human malignancies. The potentiality in co-regulation of apoptosis and autophagic cell death is implicated as the critical downstream molecular and phenotypic effectors of UBE2C in NSCLC^15^.

Both normal and malignant cells depend on autophagic response in the maintenance of organismal homeostasis^16^ in which alterations in autophagy are pathophysiologically involved in human diseases such as cancer, neurodegeneration, and immune disorders^17^. Autophagy has been implicated as a novel target in the development of cancer therapeutics for the past decade and clinically interventions are not available yet^18^.

Autophagy-mediated suppression of malignant transformation has been identified in a variety of models via a multitude of mechanisms^16^. Aged *Becn1*^+/−^ mice more likely tend to bear spontaneous malignancies than their wild-type littermates^19,20^. Systemic or focal deletion Atg5/7 promoted development of mouse benign liver adenomas or dramatically enhanced the onset of KRAS-G12D-driven pulmonary adenomas in mice^21–24^. However, autophagy also speeds tumor progression and resistance to treatment elusively^16^. In vivo studies reveal that intact tumor cell-autonomous autophagic responses are required for the initiation of therapeutically relevant anticancer immune responses in tumors developed in syngeneic immunocompetent hosts treated with immunogenic chemotherapy or radiotherapy^25–27^. The transcriptional and epigenetic regulation of autophagy is implicated in sustaining the basal autophagy^28–30^. However, the complex regulatory mechanisms controlling autophagy in both general and specific contexts remain largely inexplicit. The epigenetic and transcriptional control of autophagy is mainly triggered by upstream signaling cascades and then epigenetically modified in the nucleus.

UBE2C is encoded by the *UbcH10* gene located on human chromosome 20q13.12. UBE2C overexpression attributes to amplification of the *UbcH10* gene in some human tumors, but not in lung cancer^31^. Regulation of UBE2C activities is largely undefined yet. miR-381, as the post-transcriptional repressor of UBE2C, is downregulated in lung adenocarcinoma. Moreover, α-ketoglutarate-dependent dioxygenase alkB homolog 5 (ALKBH5) is the one of de novo demethylases for *N*^6^-Methyladenosine (m^6^A)^32–34^ in which mRNA m^6^A modification is involved on cellular processes including alterations in RNA stability, and translation efficiency^35–44^. ALKBH5 is highly expressed in lung cancer (GENT database) and hypoxia activates ALKBH5 in breast cancer cells^45^. And more, Alkbh*5* knockout significantly reduced UBE2C expression in GBM stem-like cells. Therefore, epigenetic and epitranscriptional regulation of UBE2C remain to be further mechanistically explored in lung carcinogenesis.

Activation of the oncogene UBE2C and repression of autophagy are concurrently underlying the initiation, progression, and metastasis of lung cancer. The elusive association of UBE2C with autophagy repression in lung carcinogenesis is not well elucidated and the epigenetical and epitranscriptional regulation of UBE2C has not been clearly delineated yet. These unanswered questions highlight the significance to further explore the dysregulation of UBE2C and its subsequent phenotypic repression of autophagy in lung cancer. Comprehensive molecular dissection of deregulated UBE2C-autophagy repression axis in NSCLC will render more possibilities in spotting novel molecular therapeutic targets in lung cancer.

## Results

### Aberrant activation of UBE2C in lung tumors from patients associates with adverse prognosis

**Fig. 1:**
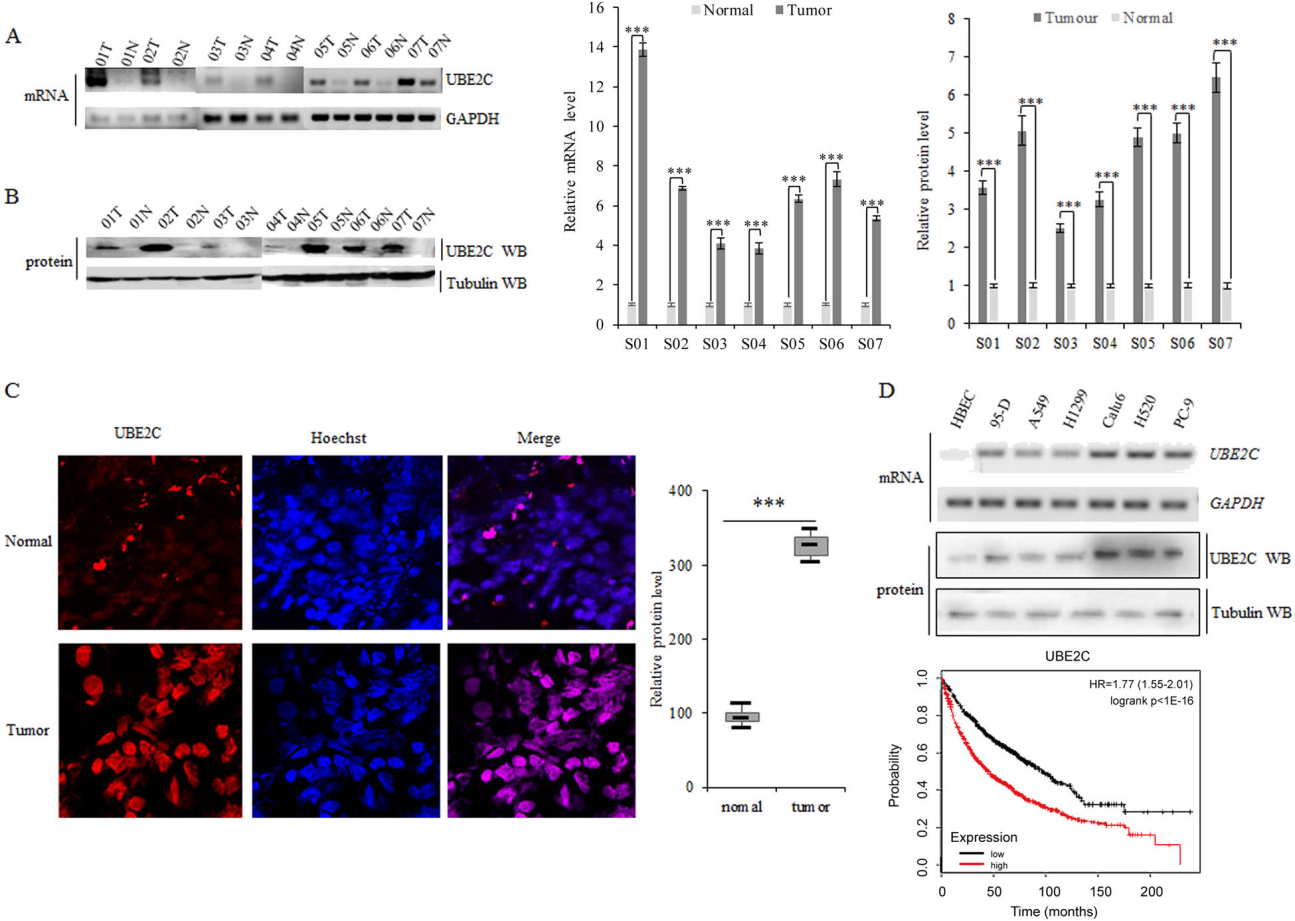
Aberrant activation of UBE2C in lung tumors from patients associated with adverse prognosis. **a** RT-PCR analysis of the endogenous mRNA levels of UBE2C in human lung cancers relative to paired normal lung tissues with densitometric quantitation. GAPDH was amplified in parallel as inner control. N and T represent normal and tumor specimens separately. **b** Immunoblotting analysis of the endogenous protein levels of UBE2C in human lung cancers relative to paired normal lung tissues with densitometric quantitation. α-Tubulin was loaded as inner control. **c** Immunofluorescent staining of UBE2C proteins showing increased UBE2C in NSCLC samples compared with their normal adjacent lung tissues as in those representative pictures and the analysis of densitometric quantitation of UBE2C positive staining (plot and box graph). **d** Immunoblotting with densitometric quantitation demonstrating more increased protein level of UBE2C in 95-D, A549, H1299, Calu6, H520 and PC-9 cells than normal cell line HBEC. **e** Kaplan−Meier overall survival (OS) curves of UBE2C (left, *n* = 1926, *P* = 1E-16 by log-rank test for significance). **P < 0.001, ***P < 0.0001 by Student’s *t* test

To profile the activated UBE2C in human lung cancer tissues with assays from RT-PCR and immunoblotting, we found that endogenous mRNA levels and protein expression of UBE2C are significantly elevated in all seven human lung cancers relative to paired normal lung tissues (Fig. 1a, b). As indicated in Fig. 1c, immunofluorescent staining additionally showed enhanced distribution of UBE2C in NSCLC samples compared with their normal adjacent lung tissues. UBE2C protein level was also significantly upregulated in all listed lung cancer cell lines (95-D, A549, H1299, Calu-6, H520, and PC-9) than normal cell line (HBEC) (Fig. 1d). The openly accessible datasets (2015 version) (http://www.kmplot.com/analysis/index.php?p=service&cancer=lung)^46–48^ were selected for screening the prognostic correlation between expression of UBE2C and survival of lung cancer patients. As the Kaplan−Meier analyses indicated, endogenous expression of UBE2C was anti-correlated with OS of patients with lung tumors in this analysis (*n* = 1926, *P* = 1E-16 by log-rank test for significance) (Fig. 1e).

### Downregulation of UBE2C reduces cell proliferation, clonogenicity, and invasive growth of NSCLC

**Fig. 2:**
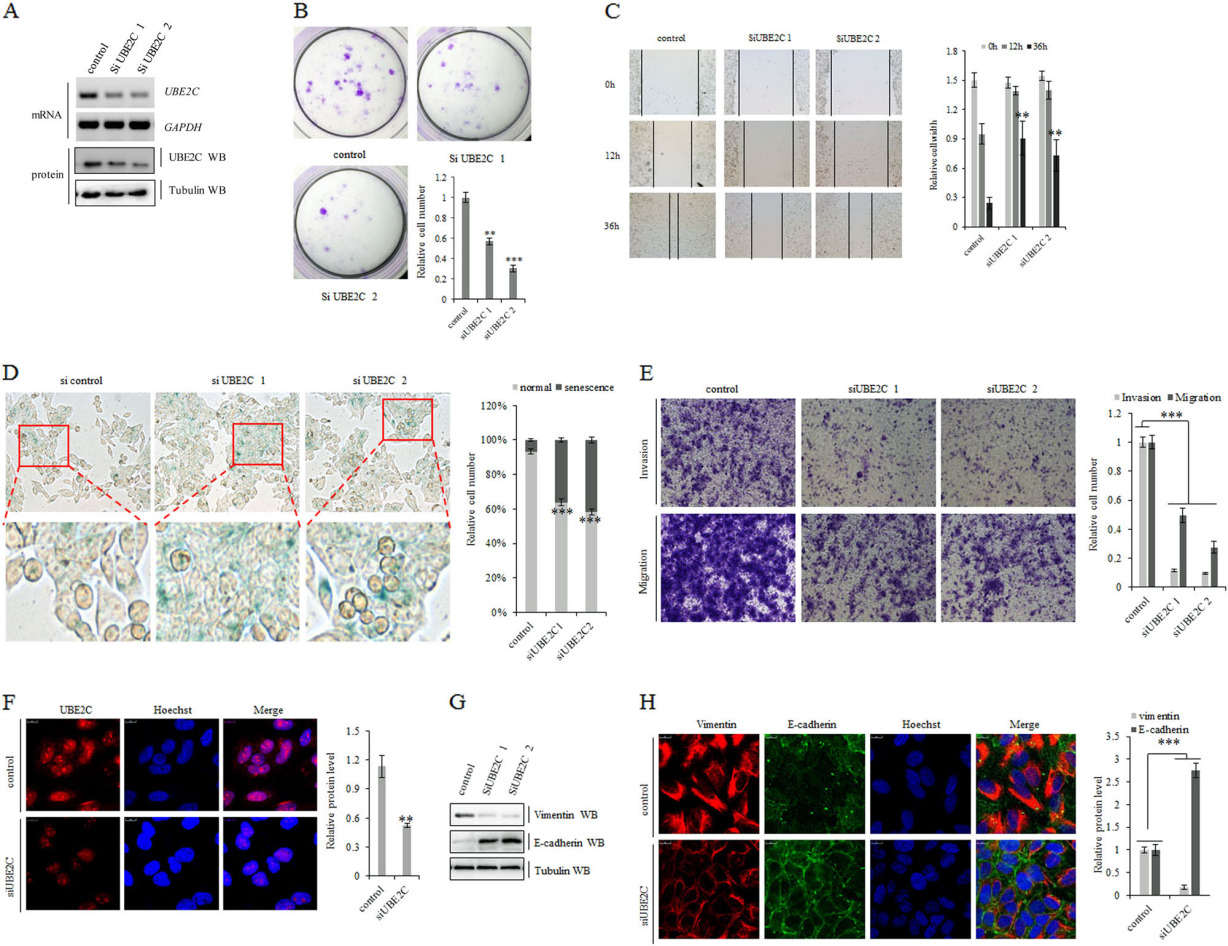
Downregulation of UBE2C reduces cell proliferation, clonogenicity, and invasive growth of NSCLC. **a** siRNA-induced specific downregulation of UBE2C at both mRNA and protein levels in A549 cells as shown in RT-PCR and immunoblotting. **b** Knockdown of UBE2C reduced clonal formation of A549 cells in the representative images of colony formation with quantitative clonogenicity assay. **c** Quantitative scratch assay showing that knockdown of UBE2C dramatically slowed cell migration in A549 cells. **d** SA-β-Gal assay showing that transient knockdown of UBE2C for 36 h significantly increased cell senescence phenotype in A549 cells. **e** Matrigel invasion assay indicated downregulation of UBE2C significantly decreased cell invasive growth and migration of A549 cells. **f**–**h** Immunofluorescent staining and immunoblotting of UBE2C and eminent EMT markers demonstrating that knockdown of UBE2C (**f**) enhanced E-cadherin (**g**, **h**) expression while repressing vimentin (**g**, **h**) in A549 cells. **P < 0.001, ***P < 0.0001 by Student’s *t* test

In previous reports, the downregulation of UBE2C could increase the expression of E-cadherin and decrease the expression of Slug, Twist, and Snail^49–51^, which are transcriptional drivers for vimentin in human cancer. Here, depletion of UBE2C by siRNA was performed to examine whether UBE2C specifically instigates the progression and metastasis of lung cancer cells. Our results showed that knockdown of UBE2C (Fig. 2a) reduced clonal formation (Fig. 2b), cell migration (Fig. 2c), and invasive growth (Fig. 2e) of A549 cells while endogenous depletion of UBE2C enhanced senescence of A549 cells (Fig. 2d). UBE2C depletion significantly elevated E-cadherin protein and mRNA levels and decreased the protein level of vimentin in A549 cells (Fig. 2f, g). As revealed in Supplementary Figure S1F, reactivation of E-cadherin and repression of vimentin induced by UBE2C deficiency were partially blocked with specific inhibition of ATG3 and LC3 in NSCLC, which suggests that UBE2C-mediated repression of autophagy is involved in execution of EMT and the consequent invasive growth of tumor cells.

### Deregulated UBE2C-autophagy repression axis in NSCLC

**Fig. 3:**
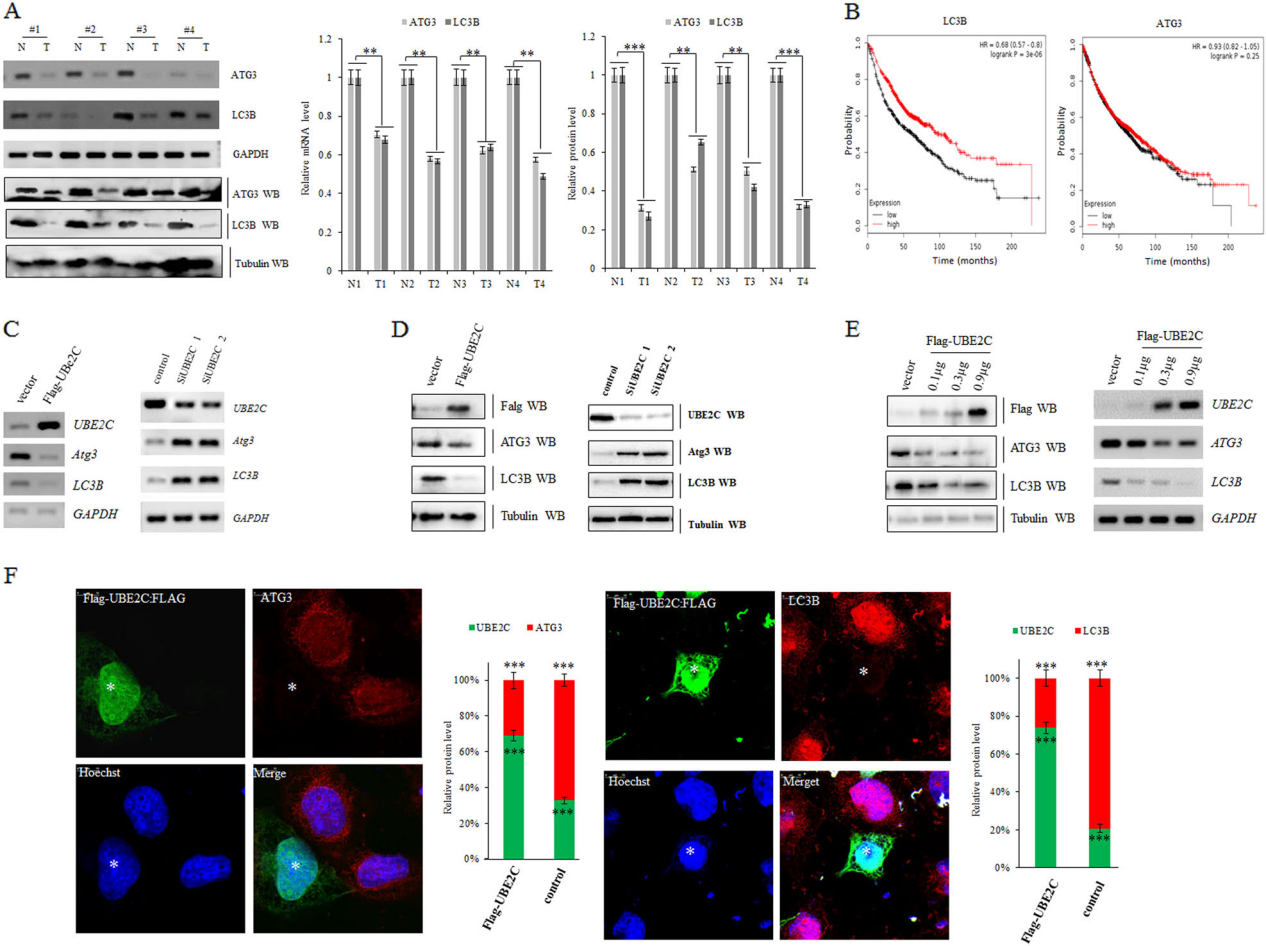
Deregulated UBE2C-autophagy repression axis in NSCLC. **a** RT-PCR and immunoblotting analysis of the endogenous mRNA levels of ATG3 and LC3B in human lung cancers relative to paired normal lung tissues with densitometric quantitation. GAPDH/α-Tubulin was amplified/loaded in parallel as inner control. N and T represent normal and tumor specimens separately. **b** Kaplan−Meier overall survival (OS) curves of LC3B (left, *n* = 1926, *P* = 3E-06 by log-rank test for significance) and ATG3 (left, *n* = 1926, *P* = 0.25 by log-rank test for significance). **c**, **d** RT-PCR and immunoblotting analysis showing that forced expression of UBE2C repressed ATG3 and LC3B at both transcription (**c**) and protein levels (**d**) while depletion of UBE2C derepressed both in A549 cells. **e** Overexpressing UBE2C dose-dependently repressed ATG3 and LC3B at both mRNA and protein levels in A549 cells. **f** Immunofluorescent staining of UBE2C and autophagy markers further confirmed that forced expression of UBE2C reduced ATG3 and LC3B levels in A549 cells. **P < 0.001, ***P < 0.0001 by Student’s *t* test

Among the Atg proteins identified for the execution of autophagy, microtubule-associated protein light chain 3B (LC3B) serves as a widely used marker for autophagosomes and LC3B-based biochemical and microscopic assays greatly facilitate the detection of autophagy. In addition, specific inhibition of the autophagy pathway can be achieved by knockout or knockdown of ATG3 with implication of ATG3 as another important molecular marker for autophagy^52^. Therefore, both LC3B and ATG3 were examined to detect autophagy in the present study. To interrogate how the autophagy is modulated and involved in lung carcinogenesis, we analyzed the expression patterns of autophagy markers ATG3 and LC3B in human lung cancers and identified that endogenous ATG3 and LC3B are significantly repressed in all four human lung cancers relative to paired normal lung tissues (Fig. 3a). Our Kaplan−Meier analyses further provided that transcriptional activity of LC3B but not ATG3 was significantly anti-correlated with OS (LC3B: *n* = 1926, *P* = 3E-06 by log-rank test for significance; and ATG3: *n* = 1926, *P* = 0.25 by log-rank test for significance) (Fig. 3b). To test whether UBE2C interferes with the activities of autophagy, we examined the responses of ATG3 and LC3B to alteration of UBE2C in A549 cells. Our in vitro assays revealed that the forced expression of UBE2C dramatically repressed the expression of ATG3 and LC3B while depletion of UBE2C activated them in A549 cells (Fig. 3c−f; Supplementary Figure S1A).

### Interruption of UBE2C-autophagy repression axis attenuates cell proliferation, clonogenicity, and invasive growth of NSCLC

**Fig. 4:**
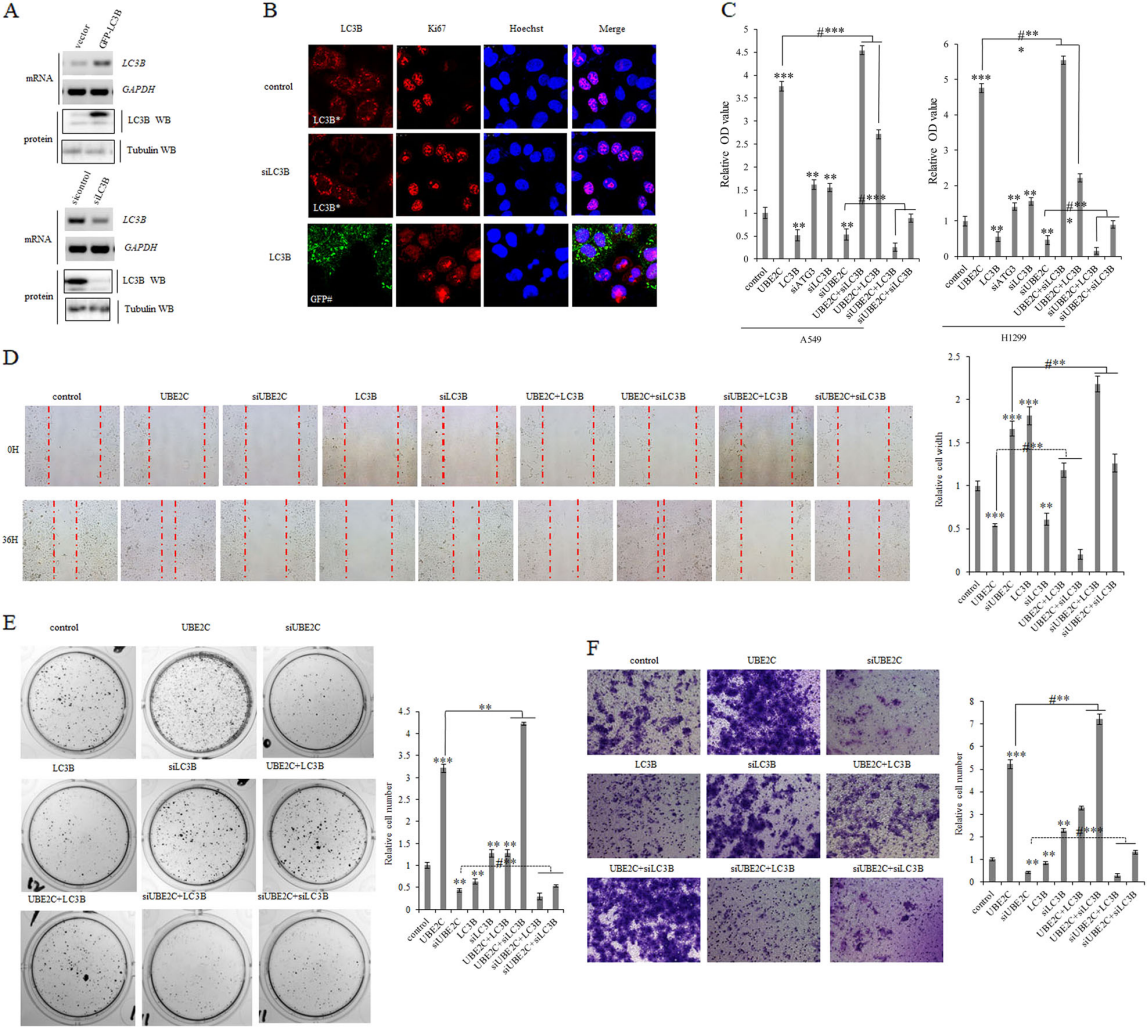
Derepression of UBE2C-autophagy repression axis attenuates cell proliferation, clonogenicity, and invasive growth of NSCLC. **a**, **b** RT-PCR, immunoblotting, and immunofluorescent staining analysis showing introduction of GFP-LC3B plasmids-induced forced expression of LC3B and siRNA-LC3B-depleted endogenous LC3B in A549 cells. **c** In vitro proliferation assay demonstrating that UBE2C overexpression or LC3B depletion drove cellular proliferation of both A549 and H1299 while UBE2C depletion or LC3B overexpression arrested growth of those cells respectively. In addition, LC3B overexpression partially blocked UBE2C overexpression-induced cellular proliferation of both A549 and H1299, and LC3B depletion compensated UBE2C depletion-induced growth inhibition of those cells. **d** Scratch assay showing that UBE2C overexpression or LC3B depletion enhanced cell migration of A549 cells while UBE2C depletion or LC3B overexpression delayed cell migration of those cells respectively. In addition, LC3B overexpression partially blocked UBE2C overexpression-induced enhancement in cell migration of A549 cells, and LC3B depletion compensated UBE2C depletion-induced reduction in cell migration of those cells. **e** Colony formation assay demonstrating that UBE2C overexpression or LC3B depletion promoted clonogenicity of A549 cells while UBE2C depletion or LC3B overexpression decreased clonogenicity of those cells respectively. In addition, LC3B overexpression partially blocked UBE2C overexpression-enhanced clonogenicity of A549 cells and LC3B depletion compensated UBE2C depletion-reduced clonogenicity of those cells. **f** Matrigel invasion assay indicating that UBE2C overexpression or LC3B depletion increased cell invasive growth of A549 cells while UBE2C depletion or LC3B overexpression decreased cell invasive growth of A549 cells respectively. In addition, LC3B overexpression partially blocked cell invasive growth of A549 cells driven by UBE2C overexpression and LC3B depletion compensated the reduction in cell invasive growth of those cells with UBE2C depletion. *P < 0.01, **P < 0.001, ***P < 0.0001 by Student’s *t* test

Since UBE2C-autophagy repression axis is evolutionarily deregulated in NSCLC and serves as a potential druggable target in lung cancer treatment, we phenotypically analyzed the consequences resulting from dysregulation of UBE2C-autophagy repression axis in lung cancer cells. Upregulation of LC3B decreased cellular proliferation marker Ki67 while endogenous depletion of LC3B activated it in both A549 and H1299 cells (Fig. 4a, b; Supplementary Figure S1D). Further in vitro proliferation assay (Fig. 4c) demonstrated that UBE2C overexpression or LC3B depletion drove the proliferation of both A549 and H1299 cells while UBE2C depletion or LC3B overexpression arrested growth of those cells respectively, in which ATG3 depletion only also stimulated cell proliferation of those cells. In addition, elevation in expression of LC3B partially blocked UBE2C overexpression-induced cellular proliferation of both A549 and H1299, and LC3B downregulation compensated UBE2C depletion-mediated growth inhibition of those cells. Our cell migration, clonogenicity, and Matrigel invasion analysis (Fig. 4d−f) revealed that UBE2C activation or LC3B knockdown promoted cell migration, clonogenicity, and invasive growth of A549 cells while UBE2C reduction or LC3B enhancement delayed cell migration, clonogenicity, and invasive growth of those cells respectively. Moreover, LC3B accumulation partially blocked UBE2C upregulation-induced enhancement in cell migration, clonogenicity, and invasive growth of A549 cells, and LC3B deficiency compensated UBE2C depletion-induced reduction in cell migration, clonogenicity, and invasive growth of those cells.

### Autophagy repression is inalienable while UBE2C is driving cell proliferation, clonogenicity, and invasive growth of NSCLC

**Fig. 5:**
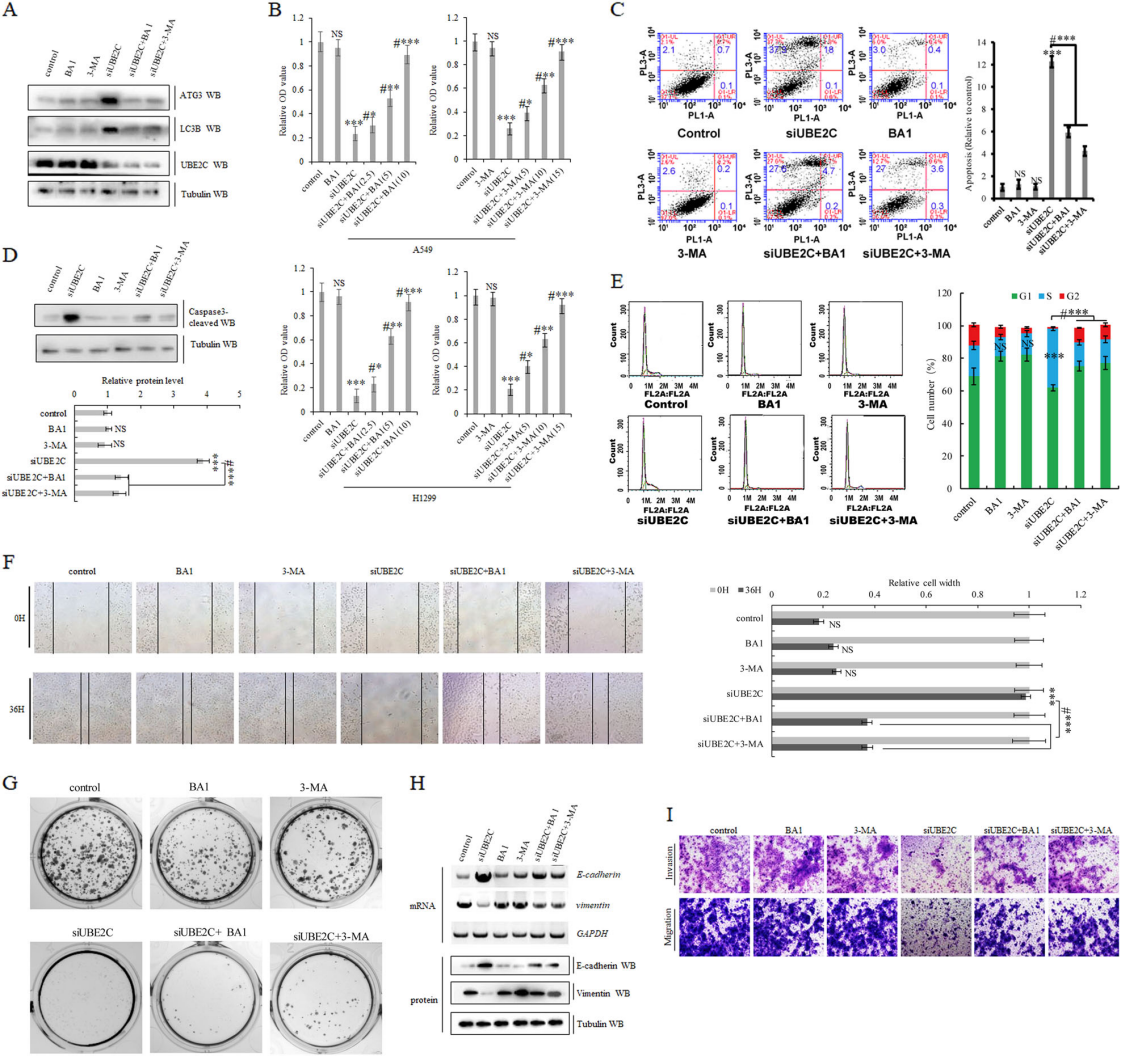
Autophagy repression is inalienable while UBE2C is driving cell proliferation, clonogenicity, and invasive growth of NSCLC. **a** Immunoblotting assay demonstrating downregulation of UBE2C significantly activated ATG3 and LC3B in A549 cells, and autophagy inhibitors BA1 or 3-MA specifically blocked the depression of ATG3 and LC3B in A549 cells with UBE2C depletion. **b** In vitro proliferation assay indicating that autophagy inhibitors BA1 or 3-MA dose-dependently blocked UBE2C depletion-induced cellular proliferation arrest of both A549 and H1299 cells respectively. **c** Flow cytometric assay showing that BA1 or 3-MA treatments decreased UBE2C depletion-induced apoptosis in A549 cells respectively. **d** Immunoblotting with densitometric quantitation illustrating that BA1 or 3-MA treatments blocked elevation of pro-apoptosis protein caspase-3 induced by UBE2C depletion in A549 cells respectively. **e** Cell cycle assay demonstrating S arrest induction by UBE2C downregulation was significantly erased with treatment of BA1 or 3-MA in A549 cells. **f** Scratch assay showing that BA1 or 3-MA treatment compensated reduction in cell migration of A549 with UBE2C deficiency. **g** Colony formation assay indicating that BA1 or 3-MA treatment partially retrieved impairment of clonogenicity in A549 cells with UBE2C depletion. **h** RT-PCR and immunoblotting analysis of EMT biomarkers demonstrating that reactivation of E-cadherin and repression of vimentin induced by UBE2C deficiency were partially blocked with BA1 or 3-MA treatment in A549 cells. **i** Matrigel invasion assay indicating that BA1 or 3-MA treatment retrieved cell invasive growth and migration arrested by UBE2C deficiency in A549 cells. ***P < 0.0001 by Student’s *t* test

UBE2C functions as one of major oncogenic drivers in initiation, progression, and metastasis of NSCLC, which rewires diverse downstream signal cascades to phenotype its oncogenic manifestation in lung carcinogenesis. Our current investigation illustrated that autophagy is negatively regulated by UBE2C in NSCLC. We asked whether autophagy repression is indispensable in UBE2C-induced lung cancer progression. Here two autophagy-specific inhibitors BA1 or 3-MA were applied to dissect the essential role of autophagy in UBE2C-mediated oncogenesis. Our results showed that activation of ATG3 and LC3B from UBE2C depletion significantly blocked both autophagy inhibitors BA1 or 3-MA while those two inhibitors did not further inhibit ATG3 and LC3B largely due to their relatively low endogenous levels in A549 cells (Fig. 5a; Supplementary Figure S1B and C). Functional exploration of autophagy repression in UBE2C-drived lung carcinogenesis not only revealed that autophagy inhibitors BA1 or 3-MA dose-dependently blocked UBE2C deficiency-induced cellular proliferation arrest of A549 and H1299 cells respectively in our proliferation assay (Fig. 5b), but also indicated that BA1 or 3-MA treatments blocked elevation of pro-apoptosis protein caspase-3 induced by UBE2C downregulation and decreased UBE2C knockdown-induced apoptosis and senescence in A549 cells respectively (Fig. 5c, d; Supplementary Figure S1E and G). Moreover, both BA1 and 3-MA significantly attenuated S arrest induction by UBE2C inhibition in A549 cells, compensated reduction in cell migration of A549 cells with UBE2C insufficiency, and at least partially retrieved impairment of clonogenicity in A549 cells with UBE2C depletion (Fig. 5e−g). Additional analyses of EMT biomarkers demonstrated that reactivation of E-cadherin and repression of vimentin induced by UBE2C repression were partially blocked with BA1 or 3-MA treatment in A549 cells. Our following invasive growth assay demonstrated that BA1 or 3-MA treatment retrieved cell invasive growth and migration arrested by UBE2C attenuation in A549 cells (Fig. 5h, i; Supplementary Figure S1F).

### Interference of UBE2C by NCTD arrests NSCLC progression

**Fig. 6:**
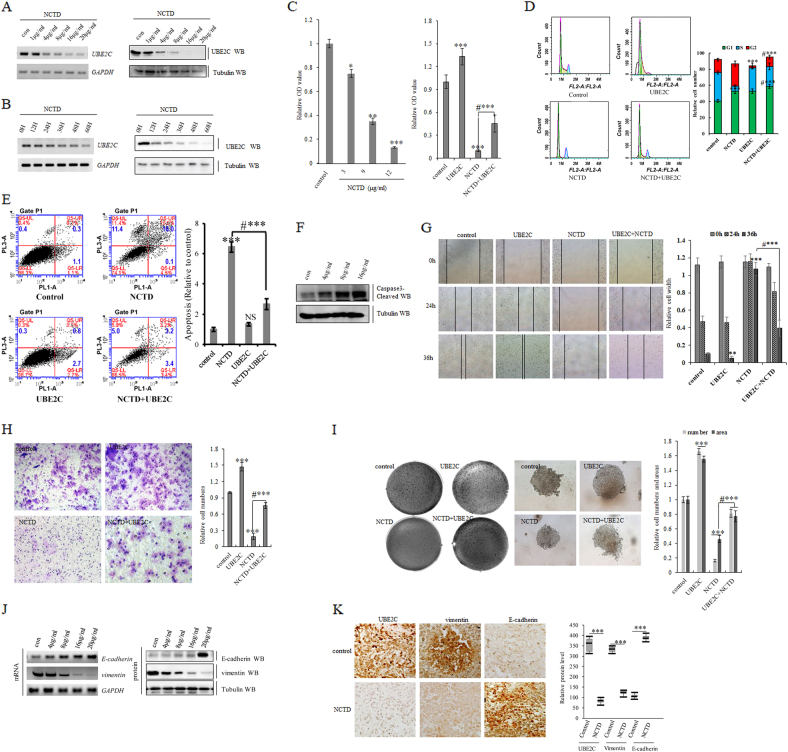
Interference of UBE2C-autophagy repression axis by NCTD arrests NSCLC progression. **a**, **b** RT-PCR and immunoblotting assay demonstrating that NCTD decreased UBE2C at both mRNA and protein level in dose-dependent (0−20 µg/ml) and time-dependent (0−60 h) manner in A549 cells. **c** In vitro proliferation assay showing that NCTD dose-dependently inhibited A549 cell growth at 3 µg/ ml or above and blocked UBE2C-induced cellular proliferation. **d** Cell cycle assay indicating NCTD at 16 μg/ml for 72 h induced significant G2 arrests and S reduction and UBE2C-induced G2 reduction cells. **e** Flow cytometric assay demonstrating that treatment of NCTD at 16 μg/ml significantly induced apoptosis in A549 cells for 72 h, in which ectopic expression of UBE2C partially blocked NCTD-induced apoptosis. **f** Immunoblotting showing that treatment of NCTD for 72 h dose-dependently increased proapoptotic protein caspase3-cleaved in A549 cells. **g** Scratch assay showing treatment of NCTD at 16 μg/ml for 24–36 h dramatically arrested cell migration of A549 cells with or without over expressing UBE2C. **h** Matrigel invasion assay indicating that treatment of NCTD at 16 μg/ml for 72 h dramatically decreased cell invasive growth and partially blocked UBE2C overexpression-induced enhancement in A549 cell invasive growth. **i** Soft agar colony formation assay demonstrating that ectopic expression of UBE2C significantly enhanced colony formation density which was blocked by NCTD at 16 μg/ml for 72 h in A549 cells. **j** RT-PCR and immunoblotting showing that treatment of NCTD for 72 h dose-dependently at 4 µg/ml or above increased E-cadherin and decreased vimentin in mRNA and protein level in A549 cells. **k** Representative pictures for immunohistochemistry staining of UBE2C, vimentin, and E-cadherin in subcutaneous tumor xenografts grown from implanted A549 cells with quantitative analysis indicating in vivo treatment of NCTD significantly decreased UBE2C and vimentin while increased E-cadherin. *P < 0.01, **P < 0.001, ***P < 0.0001 by Student’s *t* test

As a demethylated form of cantharidin, Norcantharidin (NCTD) has exhibited significant anti-tumor capacities in various human neoplasms including lung cancer with fewer side effects^47,48,53–58^. NCTD has been found to disrupt growth of a variety of human tumor cells via interfering with the cancer cell cycle progression, inducing tumor cell apoptosis, and blocking the tumor angiogenesis^59–63^. To delineate the precise molecular intervention details of NCTD in lung cancer, this study examined the selectivity of NCTD in targeting UBE2C to inhibit NSCLC progression and revealed that NCTD decreased UBE2C in dose dependent(4−20 µg/ml) and time-dependent (24−60 h) manners in A549 cells (Fig. 6a, b; Supplementary Figure S2A and C), in which concentration-dependent repression of UBE2C induced by NCTD arrested A549 cell growth and NCTD blocked UBE2C overexpression-induced cellular proliferation (Fig. 6c; Supplementary Figure S2B−E). More functional examinations of A549 cells exposed to NCTD treatment that help define the underlying mechanisms for cellular growth inhibition induced by NCTD were performed and indicated that NCTD at 16 μg/ml for 72 h interfered in cell cycle progression via enhancing significant G2 arrests/S reduction and blocked UBE2C-induced G2 reduction/S arrests in A549 cells (Fig. 6d). In addition, NCTD significantly increased apoptosis via upregulating proapoptotic protein caspase3-cleaved and reduced UBE2C ectopic expression-induced apoptosis and senescence in A549 cells (Fig. 6e, f; Supplementary Figure S2F and G) and meanwhile NCTD delayed cellular migration, invasive growth, and clonogenicity of A549 cells with or without overexpressing UBE2C to promote those carcinogenesis features (Fig. 6g−i). The EMT protein factors were specifically examined to address the responses of cellular phenotypic plasticity to NCTD. Both in vitro and in vivo studies illustrated that NCTD dose-dependently increased E-cadherin and decreased vimentin in dose-dependent manners in A549 cells and its derived xenograft tumors (Fig. 6j, k; Supplementary Figure S2H).

### NCTD targets UBE2C to depress autophagy to arrest NSCLC progression

**Fig. 7:**
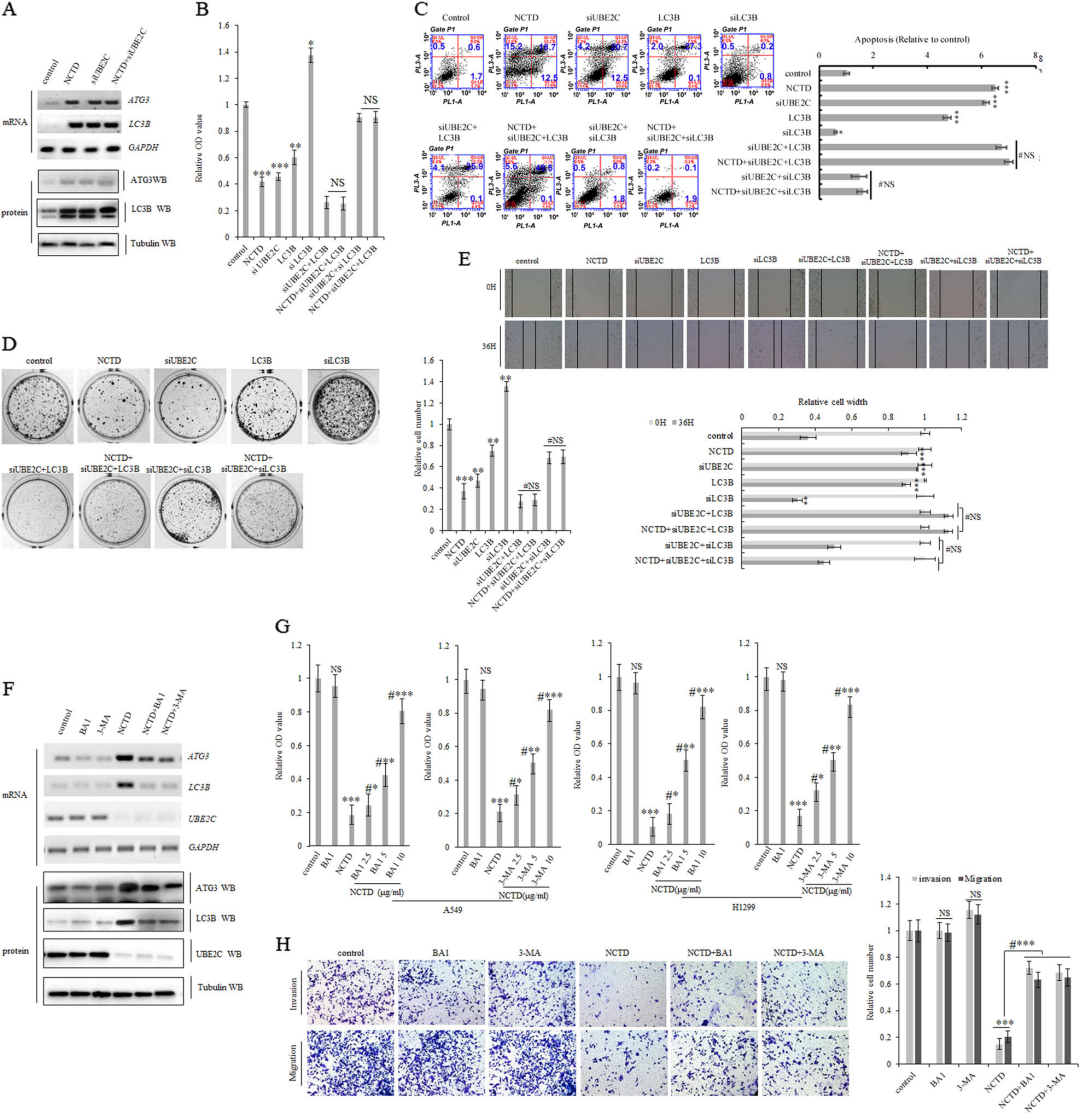
NCTD targets UBE2C to derepress autophagy to arrest NSCLC progression. **a** RT-PCR and immunoblotting analysis indicating NCTD treatment dramatically increased the mRNA and protein level of ATG3 and LC3B to the same extent as UBE2C depletion did, but this assay did not identify additive effects on expression of ATG3 and LC3B when with combination of both NCTD and UBE2C depletion in A549 cells. **b** In vitro proliferation assay demonstrating that NCTD, siUBE2C, and LC3B significantly arrested A549 cellular proliferation respectively, but this assay did not identify additive effects on cell growth when with combination of any two of NCTD, siUBE2C, and LC3B in A549 cells. **c** Flow cytometric assay demonstrating that NCTD, siUBE2C, and LC3B significantly induced apoptosis in A549 cells respectively, but this assay did not illustrate additive effects on apoptosis when with combination of any two of NCTD, siUBE2C, and LC3B in A549 cells. **d** Colony formation assay showing that NCTD, siUBE2C, and LC3B significantly reduced clonogenicity of A549 cells respectively, but this assay did not find additive effects on clonogenicity when with combination of any two of NCTD, siUBE2C, and LC3B in A549 cells. **e** Scratch assay indicating that NCTD, siUBE2C, and LC3B significantly inhibited cell migration of A549 cells respectively, but this assay did not illustrate additive effects on cell migration when with combination of any two of NCTD, siUBE2C, and LC3B in A549 cells. **f** RT-PCR and western blot analysis indicating that NCTD at 16 µg/ml for 72 h activated expression of LC3B and ATG3 at both mRNA and protein level in A549 cells in which autophagy inhibitors BA1 or 3-MA blocked NCTD-induced depression of LC3B and ATG3 in A549 cells. **g** In vitro proliferation assay demonstrating that BA1 or 3-MA does-dependently (2.5−10 unit) reversed NCTD-induced proliferation inhibition of both A549 and H1299 cells. **h** Trans-well assay indicating that NCTD at 16 μg/ml for 72 h significantly decreased invasive growth and migration of A549 cells in which treatment of BA1 or 3-MA partially retarded the NCTD-induced reduction in A549 cell invasive growth and migration respectively. *P < 0.01, **P < 0.001, ***P < 0.0001 by Student’s *t* test

Since NCTD inhibits cell growth and metastatic colonization, enhances cell apoptosis and senescence, and arrests cell cycle in lung cancer cells via selective deactivation of dysregulated UBE2C signaling networks (Fig. 6a−k) and autophagy repression is pre-required while UBE2C is driving malignant progression of NSCLC (Fig. 5a−i), we subsequently investigate whether and how NCTD interferes with the deregulated UBE2C-autophagy repression axis in NSCLC cells. First, NCTD significantly activated autophagy markers ATG3 and LC3B as UBE2C depletion did, but combination of both NCTD and UBE2C knockdown did not acquire any additive effects on expression of ATG3 and LC3B in A549 cells (Fig. 7a; Supplementary Figure S3A and B). The following proliferation assay revealed that NCTD, siUBE2C, and LC3B overexpression significantly and respectively inhibited A549 cell proliferation at the similar ranges without any additive inhibitory effects when with combination of any two of NCTD, siUBE2C and LC3B in A549 cells (Fig. 7b; Supplementary Figure S3C). To further functionally define the role of NCTD in its selective interference with UBE2C-autophagy repression axis in NSCLC cells, we measured the apoptosis, senescence, clonogenicity, and cell migration of A549 cells with treatment of NCTD, siUBE2C, LC3B ectopic expression and siLC3B or combination of them and found that NCTD, siUBE2C and LC3B significantly and respectively induced apoptosis and senescence while hampering clonogenicity, and cell invasiveness of A549 cells to the similar extent without any additive inhibitory effects when with combination of any two of NCTD, siUBE2C and LC3B in A549 cells (Fig. 7c−e; Supplementary Figure S3E-J). Moreover, autophagy inhibitors BA1 or 3-MA specifically halted NCTD-induced activation of LC3B and ATG3 and dose-dependently obstructed NCTD-mediated cell proliferation arrest and invasive growth inhibition of A549 and H1299 cells (Fig. 7f−h; Supplementary Figure S3D), in which those inhibitors did not alter the cell growth mainly owing to the existing maximum autophagy repression in NSCLC cells.

### UBE2C is post-transcriptionally and phenotypically activated in NSCLC

**Fig. 8:**
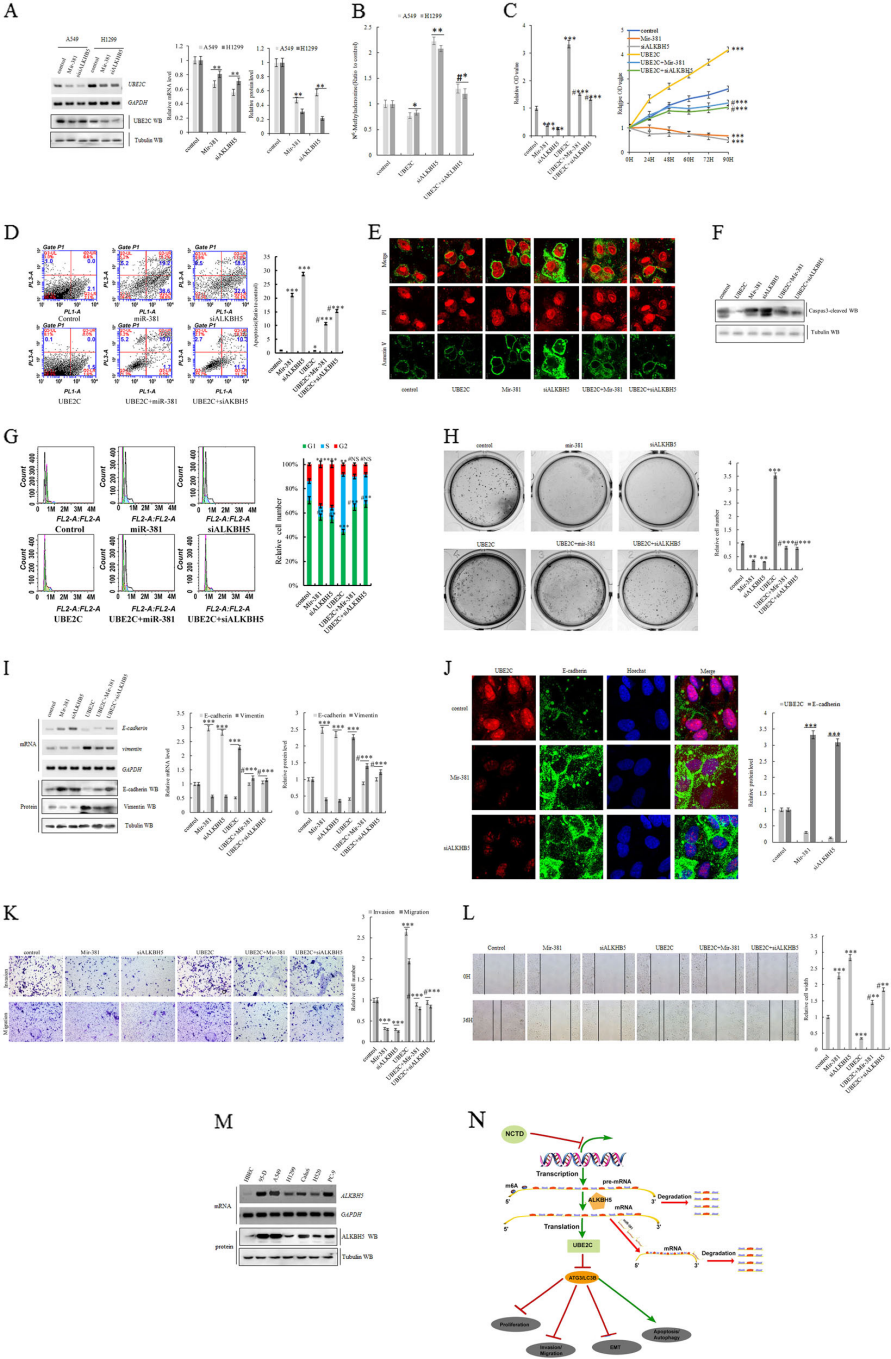
UBE2C is post-transcriptionally and phenotypically activated in NSCLC. **a** RT-PCR and immunoblotting assay of UBE2C at both mRNA and protein levels in A549 and H1299 cells treated with miR-381 mimics or siRNA specific for ALKBH5. **b** Methylated RNA immunoprecipitation analysis of m^6^A levels of pre-mRNA in A549 and H1299 cells with UBE2C overexpression or ALKBH5 knockdown or in combination. **c** In vitro proliferation assay demonstrating that miR-381 overexpression of siRNA-specific downregulation ALKBH5 decreased cell growth and arrested UBE2C overexpression-induced cellular proliferation of both A549 and H1299. **d**−**f** Flow cytometric, immunofluorescence (Annexin V), and immunoblotting (Caspase-cleaved) assay demonstrating that miR-381 overexpression and ALKBH5 knockdown significantly induced apoptosis and reduced ectopic UBE2C expression-mediated anti-apoptosis phenotypes in A549 cells for 72 h. **g** Cell cycle assay indicating treatment with miR-381 mimics or siRNA-ALKBH5-induced significant G2 arrests/S reduction and reversed ectopic UBE2C expression-induced G2 reduction in A549 cells. **h** Colony formation assay indicating that knockdown of miR-381 or ALKBH5 in A549 cells dramatically inhibited its clonal formation and arrested UBE2C overexpression-induced enhancements in its clonogenicity. **i**, **j** RT-PCR, immunoblotting, and immunofluorescence analysis of EMT biomarkers demonstrating that miR-381 overexpression or specific knockdown of ALKBH5 activated E-cadherin and repressed vimentin and resisted overexpressing UBE2C-mediated E-cadherin repression/vimentin activation pattern in A549 cells. **k** Matrigel invasion assay indicating that miR-381 overexpression or ALKBH5 downregulation in A549 cells significantly decreased the cell invasive growth and partially blocked UBE2C overexpression-induced enhancement in the cell invasive growth. **l** Scratch assay showing forced miR-381 overexpression and siRNA-specific downregulation of ALKBH5 dramatically arrested cell migration of A549 cells with or without over expressing UBE2C. *P < 0.01, **P < 0.001, ***P < 0.0001 by Student’s *t* test. **m** RT-PCR and immunoblotting assay of endogenous ALKBH5 mRNA and protein levels in HBEC, 95-D, A549, H1299, Calu-6, H520, and PC-9 cells. **n** Schematic diagram depicting the epigenetic and epitranscriptional deregulation of UBE2C-autophagy repression axis that enhances NSCLC progression. As indicated in this diagram, UBE2C transcripts negatively regulated by miR-381and destabilized by the m^6^A demethylase ALKBH5 directly and selectively represses ATG3 and LC3 which further phenotypically arrest NSCLC progression

UBE2C is abnormally upregulated in lung tumors from NSCLC patients and associated with adverse prognosis. The mechanisms governing the dysregulation of UBE2C in cancer is not defined very well so far. To identify the post-transcriptional or epitranscriptional regulators modulating UBE2C in NSCLC, we first examined the specific post-transcriptional regulators miR-381 and ALKBH5 for UBE2C in NSCLC, in which miR-381 represses UBE2C via targeting its 3′UTR and ALKBH5, one of de novo m^6^A demethylase, stabilizes the UBE2C transcripts by reducing the m^6^A methylation level inside its mRNAs. Our data showed overexpressing miR-381 and depleting endogenous ALKBH5 significantly enhanced UBE2C expression at both RNA and protein levels in A549 and H1299 cells (Fig. 8a), in which ALKBH5 deficiency-enriched m^6^A levels of pre-mRNA and UBE2C overexpression impeded ALKBH5 insufficiency-induced enrichment of m^6^A in A549 and H1299 cells as showed in methylated RNA immunoprecipitation analysis (Fig. 8b). The following in vitro phenotypical examination of interaction between those two post-transcriptional regulators and UBE2C illustrated that forced expression of miR-381 and siRNA-specific downregulation of ALKBH5 hindered cell proliferation, migration invasive growth, clonogenicity, and EMT enhancement of A549 and/or H1299 cells. Moreover, miR-381 mimic activation and ALKBH5 knockdown retarded UBE2C-mediated those phenotypic alterations in those same cells (Fig. 8c, h−l; Supplementary Figure S4). Apoptosis analysis demonstrated that elevated miR-381 and depleted ALKBH5 significantly speeded apoptosis and balked ectopic UBE2C expression-mediated anti-apoptosis phenotypes in A549 cells (Fig. 8d−f; Supplementary Figure S4). Finally, cell cycle assay further validated that treatment with miR-381 mimics or siRNA-ALKBH5 led to significant G2 arrests/S reduction and prevented ectopic UBE2C expression-induced G2 reduction in A549 cells (Fig. 8g).

Collectively, all the data above suggest that both miR-381 and ALKBH5, as the epigenetic and epitranscriptional regulators of UBE2C, are aberrantly involved in deregulation of UBE2C-autophagy repression axis which aggravates NSCLC progression. As indicated in the diagram (Fig. 8n), UBE2C transcripts negatively regulated by miR-381and interfered by the m^6^A demethylase ALKBH5 directly and selectively represses ATG3 and LC3 which further phenotypically arrest NSCLC progression.

## Discussion

The roles of aberrantly regulated UBE2C and the consequences of rewiring its downstream molecular cascades in human malignancy that initiate and sustain the carcinogenesis are less well defined. Activation of the oncogene UBE2C and repression of autophagy are concurrently underlying the initiation, progression, and metastasis of lung cancer and exploration of essential association of UBE2C with autophagy will facilitate the identification of the de novo molecular signaling network in NSCLC. Here we find that aberrant activation of UBE2C in lung tumors from patients associates with adverse prognosis and enhances cell proliferation, clonogenicity, and invasive growth of NSCLC (Figs. 1a−e, 2a−g). UBE2C selectively represses autophagy in NSCLC and depression of UBE2C-autophagy repression axis attenuates cell proliferation, clonogenicity, and invasive growth of NSCLC. Autophagy repression is inalienable while UBE2C is driving cell proliferation, clonogenicity, and invasive growth of NSCLC (Figs. 3a−f, 4a−f, 5a−i; Supplementary Figure S1). Interference of UBE2C-autophagy repression axis by NCTD arrests NSCLC progression (Figs. 5a−i, 6a−k, 7a−h; Supplementary Figure S2 and S3). UBE2C is repressed post-transcriptionally via tumor suppressor miR-381 and epitranscriptionally stabilized with enrichment and maintenance of high m^6^A level in its RNA due to downregulation of m^6^A demethylase ALKBH5 in NSCLC (Fig. 8a−m). Collectively, our results indicated that deregulated UBE2C-autophagy repression axis drives NSCLC progression that renders varieties of potential molecular targets in cancer therapy of NSCLC (Fig. 8n).

Given the aberrant activation of UBE2C in lung carcinogenesis via regulating cell cycle, apoptosis, and transcriptional process^6–8^, the mechanisms in rewiring all its downstream molecular cascades or networks in human malignancy are largely undefined yet. As one of UBE2C-targeted cellular phenotypes, apoptosis is the type-I PCD involving the activation of catabolic enzymes leading to the formation of apoptotic bodies^10–12^, which suggests that autophagy, as the type-II PCD, may be coregulated with apoptosis signaling networks in controlling the autophagic apoptosis in cancer cells^13,14^. Our result demonstrated that repression of autophagy genes associates with the elevated UBE2C expression in NSCLC (Fig. 1e). Downregulation of UBE2C depresses the ATG3 and LC3 and dramatically decreases cancer cell invasive growth, migration, and EMT phonotypic switching while enhancing apoptosis and cell cycle of NSCLC (Fig. 3c−f). Deletion of either ATG3 or LC3 can at least partially block the UBE2C-mediated enhancement in invasive growth, migration, and EMT as well as the reduction in apoptosis and cell cycle arrests in NSCLC (Fig. 4). Since the deregulated UBE2C-autophagy repression axis is one of complicated signal transduction pathways involved in the progression of lung cancer and varieties of direct downstream targets of UBE2C have been identified in driving tumor cell proliferation and invasion recently^10^, we need to further explore how UBE2C-ATG3/LC3B axis affects critical downstream target genes involved in cell proliferation and invasion. Our data revealed that upregulation of UBE2C and repression of autophagic processes rewired the expression patterns of proliferation marker Ki67, cell cycle enzymes, EMT panels, and apoptosis family members to facilitate the invasive growth of lung cancer. Considering the different etiologies and pathogenesis of NSCLC, we guessed that UBE2C might also regulate some other complicated signaling pathways differentially as its downstream targets in histological context-dependence. All our accumulated data suggest the UBE2C-repression of autophagy axis is one of pivotal aberrant activated signaling cascade in NSCLC which is an unavoidable molecular chains worthy of targeting in lung cancer.

Aberrant regulation of autophagy has been identified in human diseases such as cancer, neurodegeneration, and immune disorders. Targeting signaling molecular cascades involved in autophagy has been implicated as a target for the development of novel therapeutics for the past decade. Autophagy-mediated suppression of malignant transformation has been suggested in a variety of models and by a multitude of mechanisms^16^. Here we explored the existing status of autophagy and its upstream regulators in NSCLC and found that the depression of autophagy via downregulating UBE2C arrested the malignant phenotypic progression in lung cancer cells. It suggests that activation of autophagy genes not only alters the autophagic functions, also consequently regulates downstream signaling molecules governing the cellular proliferation, apoptosis, and metastasis-related phenotypes in NSCLC. Although autophagy has been reported to promote tumor progression and induce drug resistance through differentially context-dependent mechanisms^16^, the beneficial sides of autophagy activation were also extended in in vivo studies that autophagic activities of malignant cells are required for the initiation of anticancer immune responses in tumors established in syngeneic immunocompetent hosts treated with immunogenic chemotherapy or radiotherapy^25–27^. The transcriptional and epigenetic regulation of autophagy is implicated in sustaining basal autophagy^28–30^. However, the accurate regulatory mechanisms controlling autophagy in both general and specific contexts remain largely unknown. Our study identified UBE2C as one of the upstream transcriptional regulators of autophagy that may be further modulated by other upstream signaling cascades and epigenetic enzymes in the nucleus of NSCLC (Figs. 3a−f, 4a−f).

The mechanisms governing the regulation of UBE2C are not characterized well yet. miRNA targeting is an important post-transcriptional regulation of RNA transcripts in both normal and pathological cellular processes. miR-381, as the post-transcriptional repressor of UBE2C, is downregulated and co-exists with UBE2C activation in NSCLC, which is further validated followed by our results that showed that overexpressing miR-381 with its mimics significantly reduced the UBE2C expression in lung cancer cells (Fig. 8a). ALKBH5 functions as the one of de novo demethylases for m^6^A and regulates varieties of essential cellular processes including alterations in RNA stability, translation efficiency, alternative polyadenylation, and splicing^35–43^. We found that ALKBH5 is highly expressed in lung cancer cells (Fig. 8m), and more, Alkbh*5* knockout significantly decreased UBE2C expression in NSCLC (Fig. 8a). This study is, to our knowledge, first to report the epigenetic and epitranscriptional regulation of UBE2C in lung carcinogenesis.

Given the highlighted significance of UBE2C-autophagy repression axis in lung cancer progression, the efficacious treatments that interfere in those signaling cascades in lung cancer are lacking to date. NCTD is a demethylated form of cantharidin that has an important anticancer role in human cancers with fewer side effects^48,53–58^. NCTD not only can inhibit the proliferation of varieties of cancer cell lines and those in vivo xenografts but also present no side effects both in vitro and in vivo^55,56^. Applying NCTD as a monotherapeutic drug in clinical trials substantially benefited patients with different human malignancies^64,65^. Here, treatment application of NCTD significantly arrested NSCLC progression in dose- and time-dependent manners via dose-dependent repression of activities of UBE2C and depression of autophagy genes in lung cancer cells (Figs. 6a−k, 7a−h; Supplementary Figure S2 and S3). In addition, NCTD induced dramatic reduction in invasive growth of A549 cells implying its potential in targeting lung cancer metastasis (Fig. 7a–h; Supplementary Figure S3). Our findings strongly support the development and application of NCTD as a novel therapeutic option in the treatment of human lung cancer^47^.

This study also highlights the specific interference of transcriptional or posttranscriptional regulation of UBE2C with NCTD as the potential mechanism on how NCTD affects UBE2C (Fig. 8n). Since both miR-381-mediated posttranscriptional regulation of UBE2C and ALKBH5-induced epitranscriptional activation of UBE2C were identified as essential mechanisms governing upstream regulation of UBE2C in this study, the interaction of NCTD with those epigenetic and epitranscriptional modulators is one of pathways for NCTD-mediated specific repression of UBE2C and its downstream target genes in lung cancer. Our data cannot exclude the possibilities that NCTD targets other oncogenic drivers than UBE2C-autophagy repression axis in lung carcinogenesis.

## Materials and methods

### Cell culture and transient transfection

Human lung cancer cells HBEC, 95-D, A549, H1299, Calu6, H520, and PC-9 were cultured in RPMI-1640 medium supplemented with 10% fetal bovine serum (FBS; Hyclone, USA) in an incubator (Forma Scientific, USA) at 37 °C under a mixture of 95% air and 5% CO_2_. Plasmids were transfected with Lipofectamine^®^2000 (Thermo Fisher Scientific) reagent following the manufacturer’s instructions.

### Plasmids

Flag-tagged UBE2C and GFP-LC3B constructs were made using the pcDNA 3.1 vector (Invitrogen). Sequences encoding the Flag epitope (DYKDDDDK) was added by PCR in place of the first Met codon of the respective cDNA clones. Luciferase reporter plasmid pGL3-UBE2C was amplified and cloned into pGL3 vector (primers: forward, 5′-CCCTCGAGGGGATATGAACCTGTGTTGT-3′; reverse, 5′-CCCAAGCTTGGG-GCTCGGCTCAGCTCCTTTACGG-3′).

### Antibodies

See the Supplementary file.

### siRNAs and miRNA transfection

Transient transfection of cells was performed using Lipofectamine 2000 (Invitrogen, USA). miR-381, control, and siUBE2C were synthesized by GenePharma (Shanghai, China). miR-381, 5′-AGAGAGCUUGCCCUUGUAUAUU-3′; siUBE2C1, 5′-ACCU-GCAAGAAACCUACUCAdTdT-3′; siUBE2C2, 5′-AAUGAUGUCAGGACCAUU-CUGdTdT-3′.

### Western blotting analysis

See the Supplementary file.

### Immunofluorescent staining

See the Supplementary file.

### Cell cycle and Annexin V staining and flow cytometry

For cell cycle analysis, drug-treated cells with 80% confluence were harvested and fixed with 70% ethanol. Then cells were taken for PI staining and cell cycle was analyzed using flow cytometry. For apoptosis analysis, cells were cultured in attachment, then trypsinized and stained with PI/Annexin V (Vazyme, Apoptosis Detection Kit). Data were collected and analyzed on a BD FACSC and using FACSD via software.

### RNA isolation and reverse PCR assay

Total RNA was isolated using Trizol reagent (TransGen Biotech) and retro-transcribed into first-strand cDNA using TransScript All-in-One First-Strand cDNA Synthesis (TransGen Biotech). cDNAs were subjected to reverse PCR assay corresponding primer. GAPDH (human) served as internal control. The reverse PCR primers are as follows:

UBE2C forward primer: GGATTTCTGCCTTCCCTGAA

UBE2C reverse primer: GATAGCAGGGCGTGAGGAAC

ATG3 forward primer: AAGTGGCTGAGTACCTGACC

ATG3 reverse primer: GATCTCCAGCTGCCACAAAC

LC3B forward primer: CGCACCTTCGAACAAAGAGT

LC3B reverse primer: AGCTGCTTCTCACCCTTGTA

E-cadherin forward primer: ACCATTAACAGGAACACAGG

E-cadherin reverse primer: CAGTCACTTTCAGTGTGGTG

Vimentin forward primer: CGCCAACTACATCGACAAGGTGC

Vimentin reverse primer: CTGGTCCACCTGCCGGCGCAG

### SA-b-gal staining

Senescence-associated b-galactosidase (SA-b-gal) was detected using Senescence β-Galactosidase Staining Kit (Beyotime, C0602) following the manufacturer’s protocol.

### CCK8 analysis

See the Supplementary file.

### Wound-healing assay

See the Supplementary file.

### Clonogenicity functional assay

See the Supplementary file.

### Transwell migration assay

See the Supplementary file.

### MTT assay

See the Supplementary file.

### Immunohistochemical analysis

Some specially appointed were fixed in 4% paraformaldehyde in phosphate-buffered saline (PBS) overnight and subsequently embedded in paraffin wax. Sections were cut at 4 μm and stained with hematoxylin and eosin (HE) and immunohistochemistry (UBE2C, vimentin and E-cadherin) for histological analysis.

### m^6^A quantification

The change of global m^6^A levels in mRNA was measured by EpiQuik m^6^A RNA Methylation Quantification Kit (Colorimetric) (Epigentek) following the manufacturer’s protocol. Poly-A-purified RNA (200 ng) was used for each sample analysis.

### Human lung cancer specimen collection

All the human lung cancer and normal lung specimens were collected in Affiliated Hospital of Binzhou Medical College with written consent of patients and the approval from the Institute Research Ethics Committee. A total of seven human lung cancer samples with paired pathologically normal lungs were used for real-time PCR analysis, WB analysis, and immunohistochemistry.

## Electronic supplementary material

Supplementary file

Supplementary Figure 1

Supplementary Figure 2

Supplementary Figure 3

Supplementary Figure 4
