# Supplementary material for: Deregulation of UBE2C-mediated autophagy repression aggravates NSCLC progression
Source: Oncogenesis. 2018 Jun 13;7(6):49. doi: 10.1038/s41389-018-0054-6 (PMC6002383; doi:10.1038/s41389-018-0054-6)
Supplement: Supplementary file 1 — Supplementary file [file 41389_2018_54_MOESM1_ESM.docx]

**Materials and methods**

**Antibodies.**

Antibodies used in this study are as follows: rabbit anti-Flag (Santa Cruz 1:500), anti-UBE2C, anti-LC3B, anti-ATG3, anti-ki67 anti-E-cadherin, anti-vimentin, and anti-caspase-3 cleaved (Abcam 1:200).

**Western blotting analysis**.

Human lung cancer cells A549 and H1299 cells were transfected with corresponding plasmids and cultured for 36 h. For Western blotting analysis, cells were lysed in NP-40 buffer (10 mM Tris pH 7.4, 150 mM NaCl, 1% Triton X-100, 1 mM EGTA pH 8.0, 1 mM EDTA pH 8.0, 0.5% NP-40, and 1 mM PMSF) at 25°C for 30 min. The supernatants of the samples were diluted by 5X loading, followed by Western blotting.

**Immunofluorescent staining.**

For analysis of UBE2C subcellular localization and the protein levels of LC3B, ATG3, Ki67, caspase3-cleaved, vimentin and E-cadherin, A549 cells were grown on coverslips in a 24-well plate overnight and after 12h, treated with NCTD with 36h for analysis of UBE2C subcellular localization and various concentrations of substances with 72h for the protein levels, cells were fixed in 4% formaldehyde for 30 min and permeabilized by incubation in 2% BSA in PBS for 30 min. The coverslips were subsequently incubated with rabbit anti-UBE2C monoclonal at 1:100 dilution in PBS containing 3% BSA. Alex Fluor^®^ 594 (red) anti-rabbit monoclonal secondary fluorescence antibodies at 1:1000 dilution in PBS containing 3% BSA. DAPI (3 µg/mL) was used for nuclear staining. Images were obtained with Zeiss Axio Imager Z1 Fluorescent Microscope.

**CCK8 analysis**.

Dispense 100 µl of cell suspension (5000 cells/ well) in a 96-well plate. Pre-incubate the plate for 12 hours in a humidified incubator at 37°C, 5% CO_2_. Then add 10 µl of various concentrations of substances to the tested plate. Incubate the plate for 72 hours in the incubator. Add 10 µl of CCK-8 solution to each well of the plate and then incubate the plate for 4 hours in the incubator. Measure the absorbance at 450 nm using a microplate reader.

**Wound-healing assay.**

Cells were plated in 6-well culture plate and incubated in 5 % CO_2_ at 37°C for 12 h. Single layer confluent cells were wounded by scraping using 200P micropipette tip, then washed by PBS and incubated in RPMI-1640 containing 2% FBS with various concentrations of substances and relevant plasmid for different times. The result of this experiment was analyzed by the Olympus light microscope.

**Clonogenicity functional assay.**

For soft agar colony formation assay, virus-infected cells were added to growth medium with 0.2% agar and layered onto 1% agar beds in six-well plates. Cells were fed with 1 ml of medium every three days. The colonies were stained with 0.1% crystal violet and counted in 2–3 weeks.

**Transwell migration assay**.

Transwell migration assay was carried out with a 24-well chamber (Costar 3422, Corning Inc., Corning, NY). The lower and upper chambers were separated by a polycarbonate membrane (8 μm pore size). Cells (1x10^3^) were resuspended in RPMI-1640 medium containing free FBS in the upper chamber. The RPMI-1640 medium containing 10% FBS was added to the lower chamber. A549 cells were allowed to invade and migrate for 72h and 36h at 37˚C in a humidified atmosphere containing 5% CO_2_. The membrane was fixed in methanol for 20 min at 25˚C, and then stained with crystal violet. Cells on the upper side of the membrane were removed by PBS-rinsed cotton swabs. Cells on the lower side of the membrane were counted under an Olympus light microscope.

**MTT assay.**

Cell viability was determined by 3-(4,5-dimethylthiazol-2yl)-2,5-diphenyltetrazolium bromide (MTT) as previously described. Cells were incubated with various concentrations of substances for indicated times followed by MTT for 4h, and then 100 μL isopropanol (in 0.04 N-hydrochloric acid) was added to dissolve the formazan crystals for MTT. The absorbance was read at 570 nm using a spectrophotometer (Synergy H1, BioTek). Cell viability was calculated as the relative absorbance compared to DMSO vehicle control absorbance.

**Supplementary figure legends:**

**Figure S1. Phenotypical analysis of UBE2C-mediated autophagy gene repression in NSCLC.**

**A,** Immunoblotting and RT-PCR analysis of the protein and mRNA level of ATG3 and LC3B in A549 cells with stably expressing UBE2C or co-treatment with the proteasome inhibitor MG132. **B,** In vitro assay of cell growth effects of UBE2C overexpression / knockdown only or with LC3 over-expression/knockdown in A549 and H1299 cells. **C**, Cell growth assay indicated that BA1 and 3-MA dose-dependently retarded the growth inhibitory effect of the siUBE2C in A549 and H1299 cells by MTT assay, respectively. **D,** Immunofluorescent staining of Ki67 proteins indicated that the protein level of Ki67 was significantly decreased in A549 cells with knockdown of UBE2C using the siRNA but BA1 and 3-MA retarded the effect of the siUBE2C, respectively. **E**, Immunofluorescent staining of caspase3-cleaved proteins indicated that the protein level of caspase3-cleaved was significantly increased in A549 cells with knockdown of UBE2C using the siRNA but BA1 and 3-MA partially blocked siUBE2C-induced apoptosis, respectively. **F**, Immunofluorescent staining of E-cadherin and vimentin proteins indicated that the protein level of E-cadherin and vimentin was significantly increased and decreased in A549 cells with knockdown of UBE2C using the siRNA. Moreover, BA1 and 3-MA partially retarded the effect of the UBE2C, respectively. **G**, SA-β-Gal assay showing that knockdown of UBE2C using the siRNA significantly increased cell senescence phenotype in A549 cells but BA1 and 3-MA partially retarded the siUBE2C-induced the cell senescence, respectively. *P < 0.01**P < 0.001 ***P < 0.0001 by Student’s t-test.

**Figure S2. Interference of UBE2C by NCTD arrests NSCLC progression.**

**A**, Gel-based RT-PCR and Immunoblotting with densitometric quantitation demonstrating decreased mRNA and protein level of UBE2C by treated with NCTD and knockdown of UBE2C using the siRNA dose-dependently in A549 cells but NCTD combined with siUBE2C cannot increase the inhibition of UBE2C expression. **B**, In vitro proliferation assay demonstrating that NCTD and knockdown of UBE2C using the siRNA dose-dependently significantly arrested cellular proliferation, but NCTD combined with siUBE2C cannot increase the inhibition of cell proliferation by CCK8 assay. **C**, The effect of NCTD on the activities of UBE2C promoter was examined by luciferase reporter gene assays in A549 and H1299 cells, respectively. **D**, In vitro cell growth assay demonstrating that NCTD significantly arrested cellular proliferation in time-dependent and dose-dependent manners in A549 cells. **E**, In vitro cell growth assay demonstrating that NCTD significantly arrested UBE2C-induced A549 cell growth. **F**, SA-β-Gal assay showing that NCTD at 16μg/ml significantly increased cell senescence phenotype in A549 cells for 72h, in which ectopic expression of UBE2C partially blocked NCTD-induced senescence enhancement. **G**, Immunofluorescent staining and immunoblotting analysis of caspase3-cleaved proteins indicated that NCTD dose-dependently induced caspase3-cleaved in A549 cells. **H**, Immunofluorescent staining of E-cadherin and vimentin proteins indicated that the protein level of E-cadherin or vimentin was significantly increased or decreased in A549 cells with treatment of NCTD at 16μg/ml for 72h. **P < 0.001 ***P < 0.0001 by Student’s t-test.

**Figure S3. NCTD targets UBE2C leading to depression of autophagy to arrest NSCLC progression.**

**A**, RT-PCR and immunoblotting analysis indicated NCTD dose-dependently induced LC3B and ATG3 mRNA and protein level. **B**, Immunofluorescent staining of ATG3 and LC3B proteins indicated that the protein level of ATG3 and LC3B was significantly increased in A549 cells with treatment of NCTD at 16μg/ml for 72h and knockdown of UBE2C using the siRNA respectively, but NCTD combined with siRNA-UBE2C cannot increase the protein expression of ATG3 and LC3B. **C**, In vitro proliferation assay demonstrating that NCTD, siUBE2C and LC3B dose-dependently arrested cellular proliferation, but NCTD combined with siUBE2C or LC3B cannot decreased cell proliferation in A549 cells by CCK8 (left) and MTT assay (right). **D**, In vitro proliferation assay demonstrating that BA1 or 3-MA reversed NCTD-induced proliferation inhibition of both A549 and H1299 cells in dose-dependent and time-dependent manners. **E/F**, Immunofluorescent staining (E) and Immunoblotting (F) of caspase3-cleaved proteins indicated that the protein level of caspase3-cleaved significantly increased in A549 cells with treatment of NCTD at 16μg/ml for 72h, knockdown of UBE2C using the siRNA and stably expressing of LC3B, but NCTD combined with siUBE2C and LC3B or siLC3B cannot blocked NCTD-induced apoptosis. **G**, SA-β-Gal assay showing that NCTD at 16μg/ml for 72h, siUBE2C and LC3B significantly increased cell senescence phenotype in A549 cells, in which NCTD combined with siUBE2C and LC3B or siLC3B cannot blocked NCTD-induced senescence enhancement. **H/I**, Immunoblotting (H) and immunofluorescent staining (I) of caspase3-cleaved proteins indicated that the protein level of caspase3-cleaved significantly increased in A549 cells with treatment of NCTD at 16μg/ml for 72h, in which BA1 and 3-MA respectively retarded the effect of the NCTD-induced upregulation of caspase3-cleaved. **J,** SA-β-Gal assay showing that NCTD significantly increased cell senescence phenotype at 16μg/ml for 72h in A549 cells, in which BA1 and 3-MA blocked NCTD-induced senescence enhancement, respectively.

**Figure S4.** Immunofluorescent staining of caspase3-cleaved, KI67 and eminent EMT markers demonstrating that miR-381 overexpression or ALKBH5 knockdown enhanced caspase3-cleaved expression and repressed both Ki67 and vimentin in A549 cells as well as mitigated the UBE2C overexpression-induced induction of caspase-3 cleaved and repression of both Ki67 and vimentin in these cells.
