# Supplementary figures and images for: Deregulation of UBE2C-mediated autophagy repression aggravates NSCLC progression
Source: Oncogenesis. 2018 Jun 13;7(6):49. doi: 10.1038/s41389-018-0054-6 (PMC6002383; doi:10.1038/s41389-018-0054-6)

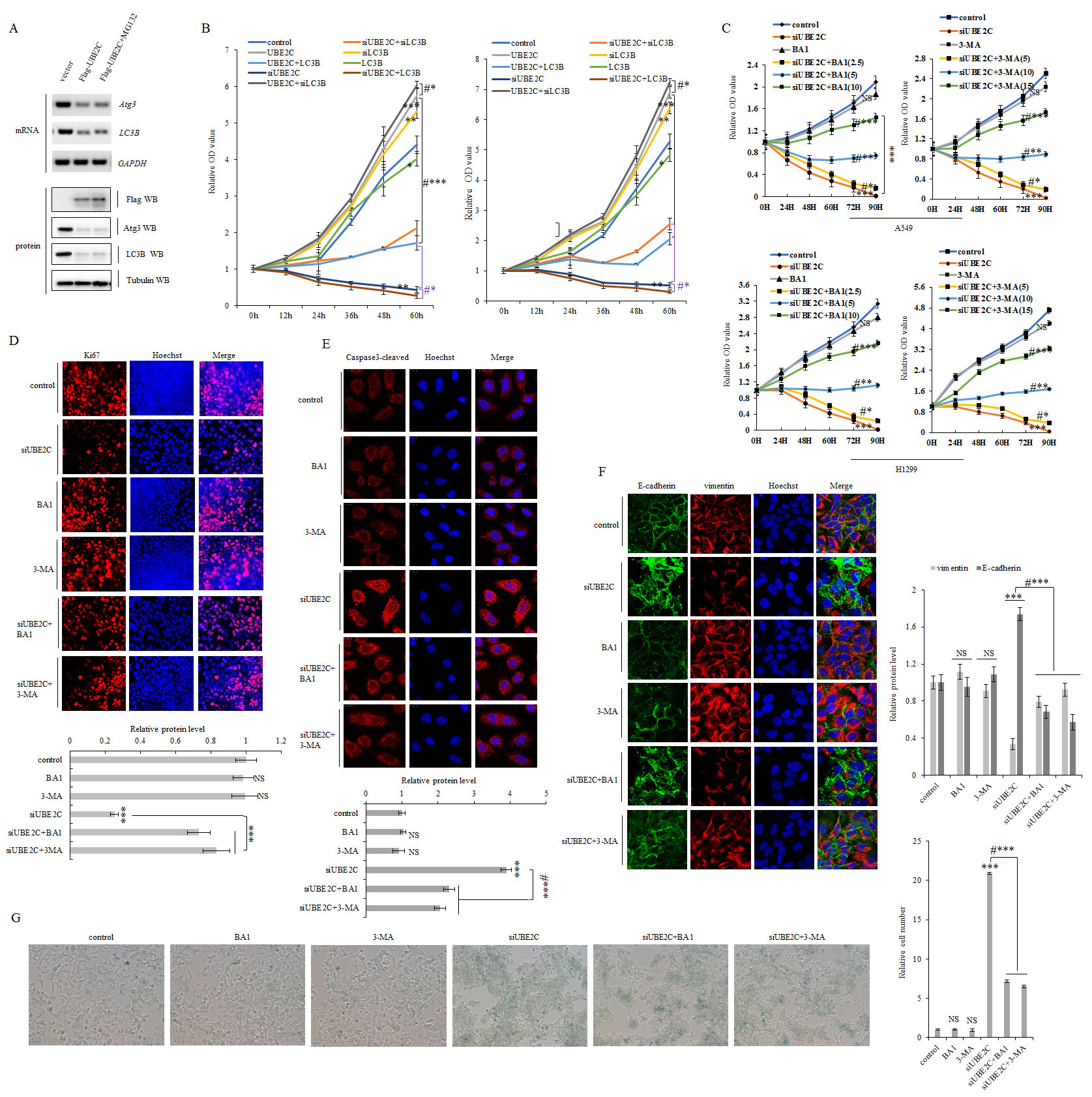

Supplement: Supplementary file 2 — Supplementary Figure 1 [file 41389_2018_54_MOESM2_ESM.jpg]

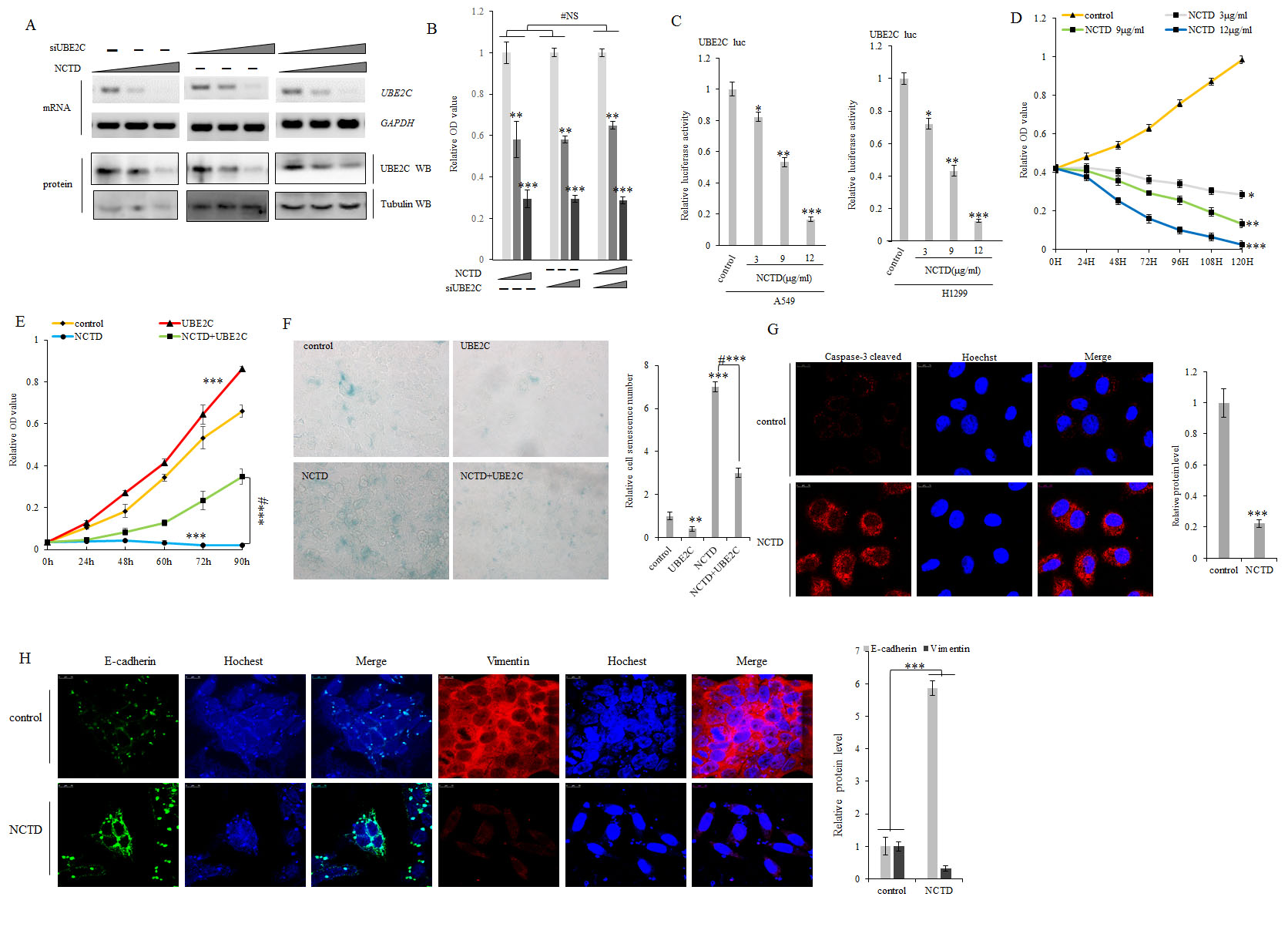

Supplement: Supplementary file 3 — Supplementary Figure 2 [file 41389_2018_54_MOESM3_ESM.jpg]

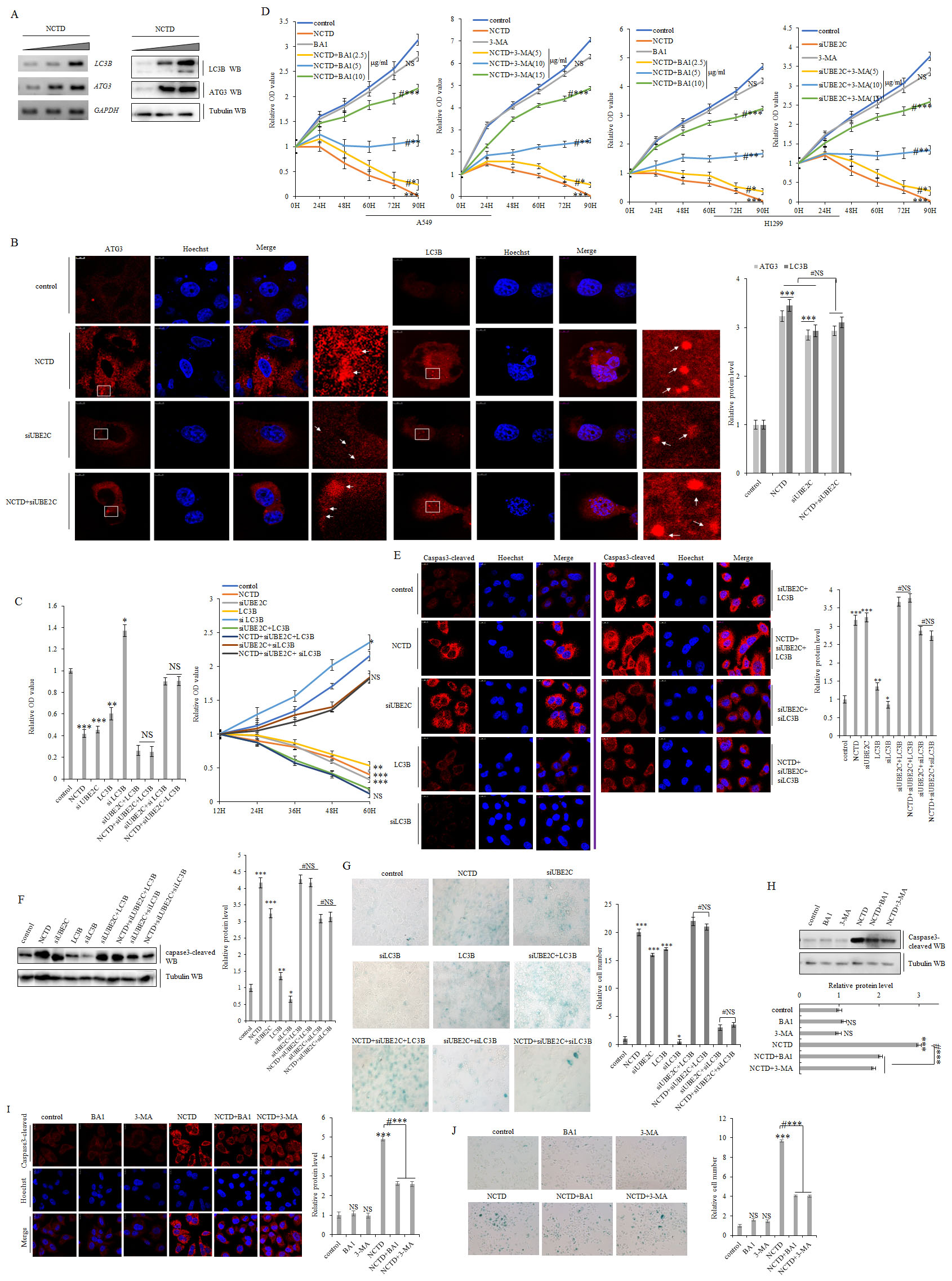

Supplement: Supplementary file 4 — Supplementary Figure 3 [file 41389_2018_54_MOESM4_ESM.jpg]

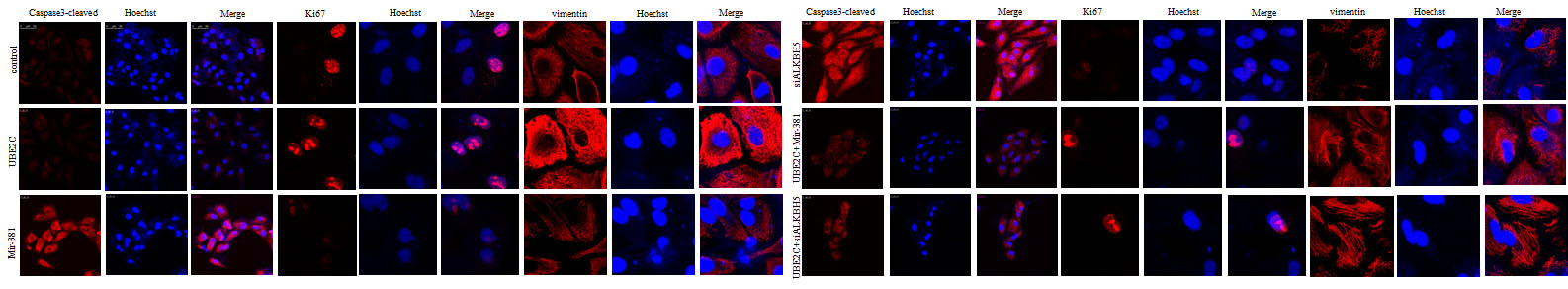

Supplement: Supplementary file 5 — Supplementary Figure 4 [file 41389_2018_54_MOESM5_ESM.jpg]
